# Gamma-ray shielding effectiveness, optical, mechanical, dielectric properties of nanofiller-reinforced PVA/PVP polymeric blend nanocomposites

**DOI:** 10.1038/s41598-024-76397-8

**Published:** 2024-11-10

**Authors:** Aya El Rahman, H. S. Metwally, N. Sabry, M. I. Mohammed

**Affiliations:** 1 Nanoscience Laboratory for Environmental and Bio-medical Applications (NLEBA), Green Research Laboratory (GRL), Department of Physics, Faculty of Education, Ain Shams University, Roxy, Cairo, 11757 Egypt; 2 Department of Physics, Faculty of Education, Ain Shams University, Roxy, Cairo, 11757 Egypt

**Keywords:** Fe_2_O_3_/(PVA-PVP) nanocomposites, XRD/FTIR analysis absorption spectroscopy, Optical filter, Conductivity mechanism, Radiation shielding, Materials science, Nanoscience and technology, Physics

## Abstract

The aqueous solution cast method was used to create the biodegradable polymer nanocomposite (PNC) films from a blend of poly (vinyl alcohol) PVA and poly (vinyl pyrrolidone) PVP (70/30 wt %) and Fe_2_O_3_ nanoparticles (NPs). These PNC films were characterized using X-ray diffraction, scanning electron microscopy SEM, Fourier transform infrared spectroscopy FTIR, and ultraviolet-visible spectroscopy. XRD and FTIR results indicate that Fe^+ 3^ NPs interact with the host polymer. Optical, electrical, mechanical, and radiation shielding measurements were performed on the PNC films. From the optical measurements, the indirect optical band gap drops from 4.86 eV for the pure blend to 4.26 eV at the greatest NPs concentration. Optical limiting characterization shows that the output power of He-Ne and solid-state green laser beams is reduced from 22.98 to 3.6 mW and 6.59 to 1.4 mW, respectively, when the Fe_2_O_3_ NPs content in the blend matrix is increased to 6 wt %. The NGCal software was utilized to calculate nuclear radiation shielding properties. The findings demonstrated that when the concentration of Fe_2_O_3_ rose, the PNC films half-value layer and mean free path decreased. Mechanical measurements demonstrate that increasing the Fe_2_O_3_ content significantly improves nanocomposite films’ yield and tensile strength. Tensile strength is measured at 27.03 MPa for the composite film containing 6 wt % Fe_2_O_3_, which is significantly higher than the 8.66 MPa of the pure (PVA-PVP) film. Compared to the other samples under examination, the 6 wt % Fe_2_O_3_ sample yielded the best results (based on the analyzed optical, electrical, mechanical, and radiation shielding properties).

## Introduction

The hybrid polymer nanocomposites are a novel material type exhibiting various physical and chemical properties. These nanocomposites have recently attracted the attention of researchers due to their real promise for a wide range of applications in environmental solutions and resolving multiple environmental concerns. The most challenging feature of PNCs is the complex interplay between nanoparticles and polymer metrics^1,2^. The small dimensions lead to large specific surfaces, highlighting the importance of the interactions between polymer and nanoparticle^3^. Investigating the interpolation process between nanoparticles and polymer bases is crucial to achieving mechanical, thermal, dielectric, optical, and electrical properties^4,5^ .

Blending polymer materials is an effective strategy to control mechanical and physical properties^6,7^. The type and content of polymers, number of phases, morphology, and strength of the polymer/polymer interface are key elements that impact the system’s final structure^6,8^. While some aspects can be modified during the blending process (e.g. blend morphology), others are determined by the inherent properties of the ingredients, such as phase miscibility, which impacts the polymer/polymer interface. Given the disadvantages of a weak interface, it is critical to use a specific technique to improve the region’s features. Numerous studies have assessed the various options, with compatibilizers (coupling agents) being the most widely known application. However, the composition of these molecules can aid in the creation of a strong contact. Recent research indicates that using polymer nanocomposites can improve interfacial properties^8,9^. Due to their superficial active chemical sites, nano-fillers form a strong polymer/particle interphase with surrounding polymers. This allows stress (shear, tensile, etc.) to easily transfer from the bulk to the reinforcing phase^10^. It is worth noting that the polymer/particle interphase has a considerable influence on the mechanical properties of polymer nanocomposites^10^. However, the mechanical parameters of the interphase area differ greatly from those of the surrounding polymer bulk, requiring the adoption of particular methodologies to assess its reaction to applied stress^10,11^.

PVA, or polyvinyl alcohol, a semicrystalline polymer having various applications, is one of the polymers used in blending. It has excellent film and fiber forming, water solubility, nontoxicity, biocompatibility, superior chemical resistance, and good mechanical characteristics^12,13^. Semicrystalline polymeric materials have regions of structural order and disorder, known as crystalline and amorphous regions, which are separated by regions of intermediate order^14^. Polyvinyl pyrrolidone (PVP), is the second used polymer. PVP is an amorphous polymer with a high *T*_*g*_ that forms various types of complexes with numerous inorganic salts given the existence of the rigid Pyrrolidone group. Also, it is one of the conjugated polymers with moderate electrical conductivity, easy processability, and good environmental stability. Its uses are numerous and include electrochemical devices (batteries, displays, etc.)^15^. The 70 PVA/30 PVP composition was chosen due to its stability and the highest optical property value among the other compositions^16,17^.

Extensive studies have been conducted on the properties and possible applications of metal oxides due to their key role in several applications such as sensors, LEDs, photovoltaics, optoelectronics, detectors, and photocatalysts^18^. Iron oxide (Fe_2_O_3_) has attracted the most interest because of its exceptional magnetic characteristics, resistance to corrosion, chemical and thermal stability, and environmental friendliness^19,20^. Compared to other metal oxides, such as rare-earth metal oxides, which are frequently more costly, less accessible, and less environmentally friendly, these qualities make Fe_2_O_3_ extremely beneficial. Fe_2_O_3_ has strong magnetic characteristics that make it excellent for applications in magnetic storage, sensing, and spintronics, where rare-earth metal oxides may not perform as effectively. These benefits are in addition to its economic and environmental benefits^21^. Fe_2_O_3_’s optical bandgap of 2.1 eV makes it an excellent choice for optoelectronics and solar energy applications, adding to its versatility^22^. Additionally, the range of synthesis techniques for Fe_2_O_3_, including spray pyrolysis, electron-beam, chemical deposition, thermal evaporation, and sol-gel, permits exact control over its characteristics, allowing it to satisfy the unique requirements of many applications^23^. Additionally, Fe_2_O_3_ has demonstrated efficacy in radiation shielding, especially in gamma-ray attenuation because of its large atomic number, providing better protection than certain rare-earth oxides^24^. Fe_2_O_3_ is a preferred choice for improving the performance of polymeric blend nanocomposites because of its multifunctionality, cost-effectiveness, and environmental advantages. This sets it apart from other metal oxides in many high-tech applications.

Effective radiation shielding materials are becoming more and more necessary due to the growing use of ionizing radiation in industrial, medicinal, and research applications^25^. Traditional shielding materials, like lead, have a lot of disadvantages, such as toxicity, high density, and environmental concerns, even if they provide good protection^25^. These drawbacks highlight the critical need for the development of effective, non-toxic substitute shielding materials. Because polymeric composites are lightweight, flexible, and adjustable, they have become attractive options for radiation shielding, especially when reinforced with nanofillers^25–27^ Among these, blends of polyvinyl alcohol (PVA) and polyvinylpyrrolidone (PVP) have drawn interest due to their superior mechanical strength, biocompatibility, and film-forming capacity. Nevertheless, PVA/PVP blends have not yet reached their full potential as gamma-ray shielding materials, particularly when nanofillers are included.

Previous studies have mostly concentrated on the mechanical, optical, and dielectric characteristics of polymeric nanocomposites, with comparatively little attention paid to their efficacy in shielding against gamma radiation. Additionally, there hasn’t been much research done on how the concentration of nanofiller affects the overall performance of PVA/PVP blends, especially in terms of achieving a balance between mechanical strength and radiation shielding ability. Few research teams have investigated the shielding capabilities of polymer nanocomposite materials recently. Includes them, K. Srinivasan et al.^28^ investigated PVA, which contains 0.5% metal oxide (Fe_3_O_4_), and its efficacy in radiation shielding. Based on its relative shielding parameters, their result provides superior shielding properties^28^. A nanocomposite based on magnetite and PVA was developed by Badawy et al.^29^. According to their findings, the performance of the PVA/magnetite nanocomposite film as a radiation shield was enhanced. It might be because of PVA and superconducting characteristics at room temperature, which causes saturation magnetization *Ms* to be lower than that of pure magnetite^29^. This study aims to fill this research gap by methodically assessing the mechanical strength, optical characteristics, dielectric behavior, and efficacy of gamma-ray shielding of nanofiller-reinforced PVA/PVP polymeric blend nanocomposites. By doing this, we hope to gain a thorough understanding of how nanofillers can improve the PVA/PVP blends’ multifunctional characteristics and ultimately aid in creating radiation shielding materials that are effective, safe, and friendly to the environment.

## Experimental work

### Materials

The compounds used in this study were 98% ferric nitrate (Fe (NO_3_)_3_.9H_2_O), Citric acid (C₆H₈O₇), PVA which has a molecular weight of 1,25,000 g.mol^− 1^ and is of a highly analytical grade, PVP and has a molecular weight 40,000 g.mol^− 1^ were purchased from (Alpha Chemical India). None of the compounds were further refined before use. Each sample was processed using deionized water. The steps involved in creating α-Fe_2_O_3_ nanoparticles are shown in the schematic representations. respectively.

### **Preparation of samples**

**Scheme 1:**
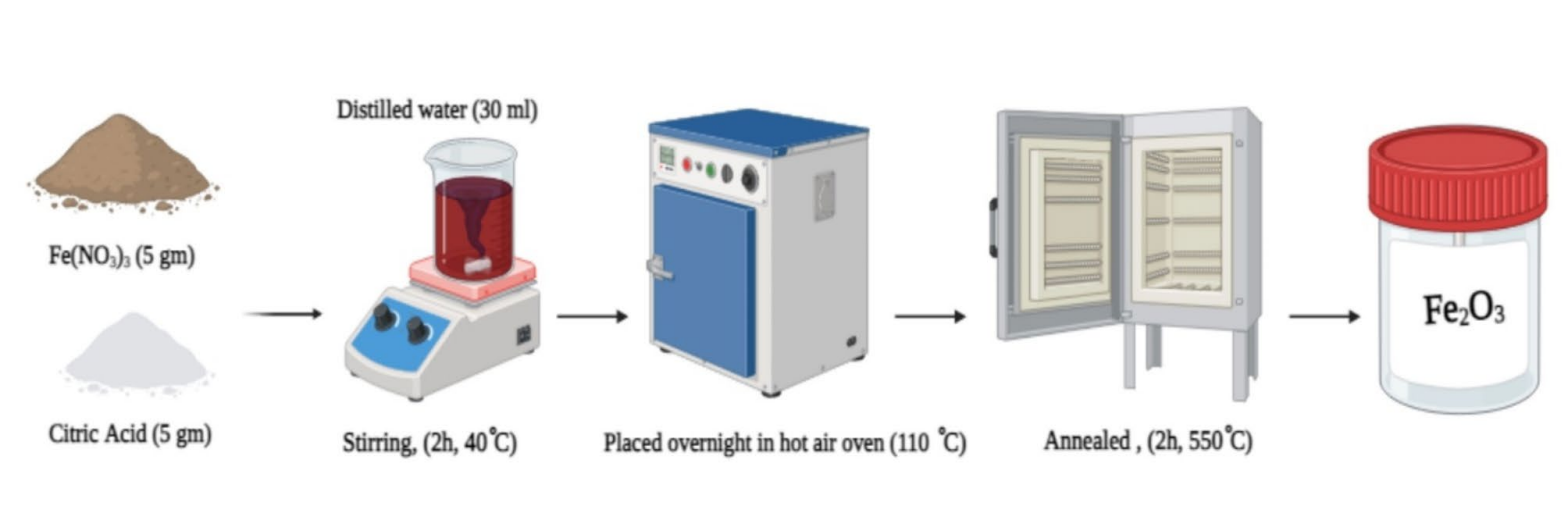
Fe_2_O_3_ nanoparticles preparation.

The sol-gel method was utilized to create Fe_2_O_3_ nanoparticles, as follows. First, dissolve 5 g each of citric acid (C₆H₈O₇) and high-grade ferric nitrate (Fe (NO_3_)_3_.9H_2_O) in 30 mL of DD water for 2 h stirring with a magnetic stirrer at 40 °C. After that, the sol was baked at 110 °C for 12 h. Ultimately, the gel was calcined at 550 °C for 2.0 h to produce Fe_2_O_3_ NPs (see scheme 1).

Solution casting was the technique used to construct PVA/PVP films, which have 70% PVA and 30% PVP composition. 30 g of PVA were fully dissolved in 1 litter of DD water at 60 °C with continual stirring, resulting in a clear and homogenous solution. 3 g of PVP was dissolved in 100 mL of DD water, and the PVA solution was then blended. Subsequently, the blend of PVA and PVP solution was stirred at room temperature for two hours. The necessary quantities of Fe_2_O_3_ NPs (0.0, 0.06, 0.3, 0.6, 3, and 6) wt % were added. To prevent aggregation the solution was mixed using an ultrasonication homogenizer treatment system for five minutes at 110 watts. To ensure that all the solvent was gone, the combined mixture was placed on Petri plates and dried in an oven at 40 °C for seven days.

**Scheme 2:**
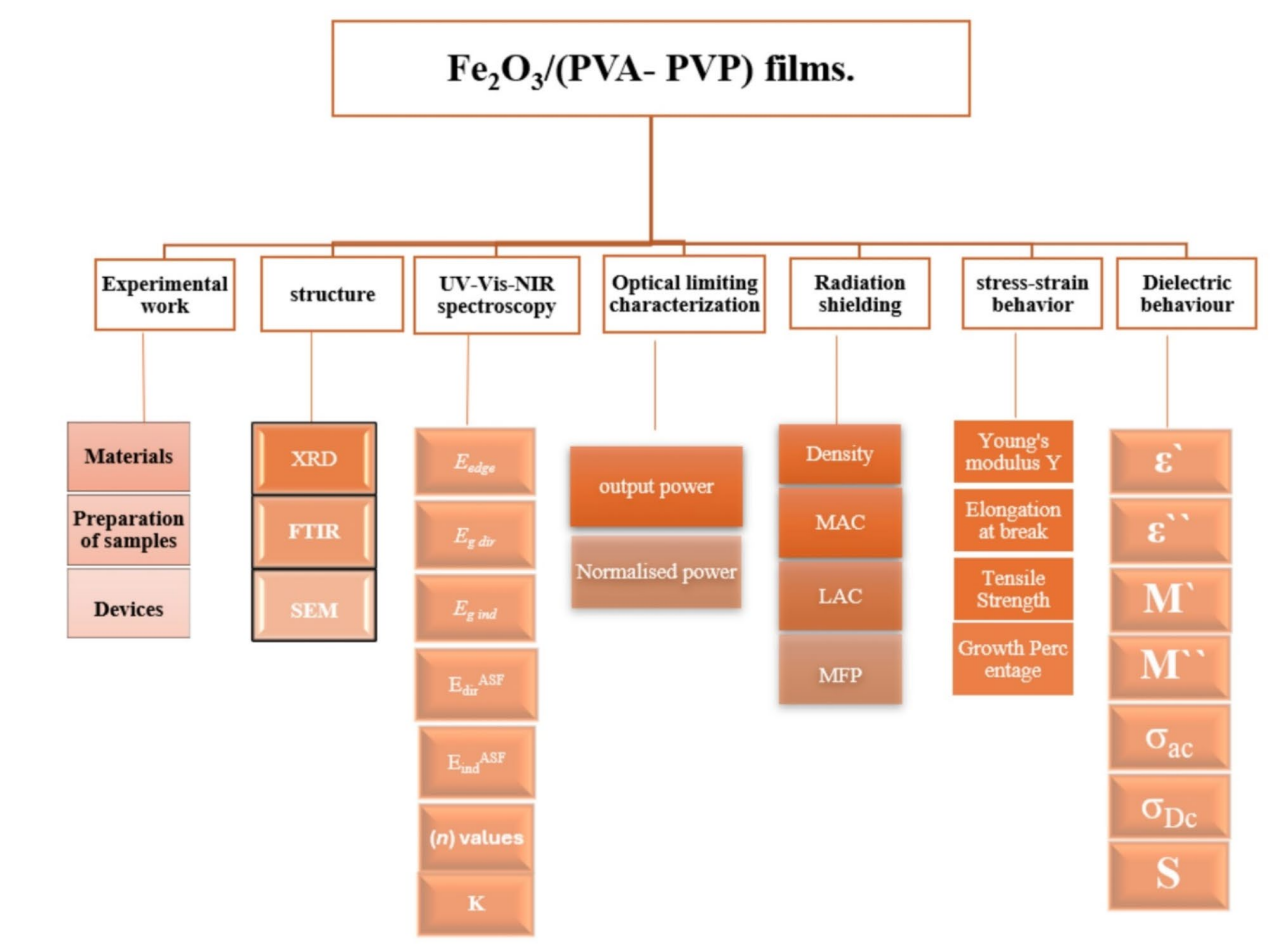
Flow chart of the measured experimental parameters.

The weight% of Fe_2_O_3_ NPs in the host PVA/PVP polymeric blend is determined using the following formula:

1 $$xwt.\% =\frac{{{w_d}}}{{{w_d}+{w_p}}} \times 100$$

where $\:{w}_{d}$ indicates the weight of the α- Fe_2_O_3_ NPs and $\:{w}_{P}$ indicates the weight of the PVA/PVP polymeric blend. All the measurements in this article were performed on these samples see Scheme 2.

### Devices

Lab XRD-6000 Shimadzu diffractometer uses the CuK_α_ source (*λ* = 1.54056 Å) Monochromatic radiation method was used to detect X-ray patterns to determine the crystallographic structure of the polymeric nanocomposite used in the films at a step of 0.02^°^, the XRD patterns were recorded between 10^°^ and 80^°^. Investigating the structural alterations and nanofiller interaction with the host PVA/PVP blend was done using the Fourier transform infrared (FT-IR; Shimadzu, IRAffinity-1 S) technology in the 400–4000 cm^–1^ wavenumber range.

A 20 kV scanning electron microscope (SEM) model (JSM-6360) was used to analyze the materials’ surface morphology. The absorbance *A*(*λ)* and transmittance *T*(*λ*) of the studied polymeric composite films were measured in the UV-Vis and NIR regions using JASCO spectrophotometers V-570. Using two sources of 632.8 nm-wavelength He-Ne laser beam and 532 nm-wavelength solid-state laser diode beam to acquire the normalized power of the nanocomposite films, the optical limiting effect for PVA-PVP/Fe_2_O_3_ composite polymeric films was investigated. An optical measuring device and a photodetector (Model: Newport 1916-R) were used to determine the input/output power^30^.

The radiation attenuation properties of polymeric nanocomposite films were investigated using the NGCal software^31^. This software was used to calculate radiation shielding parameters (the linear and mass attenuation coefficients, half and tenth value layers, mean free-path, and effective atomic number) over a wide range of ionizing radiation energy (30 keV − 15 MeV)^31^.

## Results and discussion

### XRD, FTIR, and SEM analysis

**Fig. 1:**
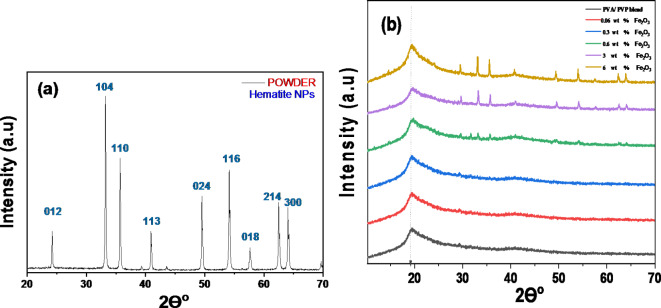
XRD patterns of 2θ range (20° to 70°) (a) Fe_2_O_3_ NPs, (b) Fe_2_O_3_/(PVA- PVP) films.

The structural properties of the Fe_2_O_3_ NPs were assessed using XRD, as demonstrated in Fig. 1a, which shows the hematite’s X-ray diffraction pattern after it has been annealed for three hours at 550 °C. There are multiple diffraction peaks in the sample’s XRD pattern (012), (104), (110), (113), (024), (116), (018), (214) and (300) attributed to the rhombohedral structure of α-Fe_2_O_3_’s diffraction planes contained *2θ* values at (24.12^o^, 33.18^o^, 35.67^o^, 41^o^, 49.6^o^, 54.15^o^, 57.6^o^, 62.4^o^, 64.11^o^) (JCPDS card No: 79–0007). The absence of secondary phases suggests that the synthesized powder is pure and consists of a single phase of α-Fe_2_O_3_. Using Scherer’s formula, the crystallite size (*D*) of Fe_2_O_3_ NPs was determined^32–34^:

2 $$D=\frac{{K\lambda }}{{\beta \cos \theta }}$$

where *K*, *λ*, *θ* and *β* are a constant, the X-ray wavelength, the reflectance angle, and the full width at half maximum respectively. The crystallite size (*D*) had an average value of 13 nm.

Figure 1b displays the XRD patterns for Fe_2_O_3_ / (PVA/PVP) nanocomposites. The PVA semi-crystalline polymer is responsible for the diffraction peak at 19.23° seen in pure PVA/PVP blend^35^ .

In addition to the peak of the amorphous blend at *2θ* = 19.23°, distinctive diffraction patterns of the Fe_2_O_3_ NPs were seen. These reflections confirm the presence of the Fe_2_O_3_ NPs in the blend matrix. Increasing the concentration of Fe_2_O_3_ NPs resulted in peaks in XRD patterns, especially at 2θ = 33.18 ^o^, 35.67 ^o^. At larger concentrations, nanoparticles may disrupt the normal arrangement of polymer chains(*2θ* = 19.23°), resulting in a loss in crystallinity due to increased matrix disorder. The produced Fe_2_O_3_ doped PVA/PVP polymeric nanocomposite films’ diffraction data in this work closely matched those of earlier published studies^16,17,36,37^.

**Fig. 2:**
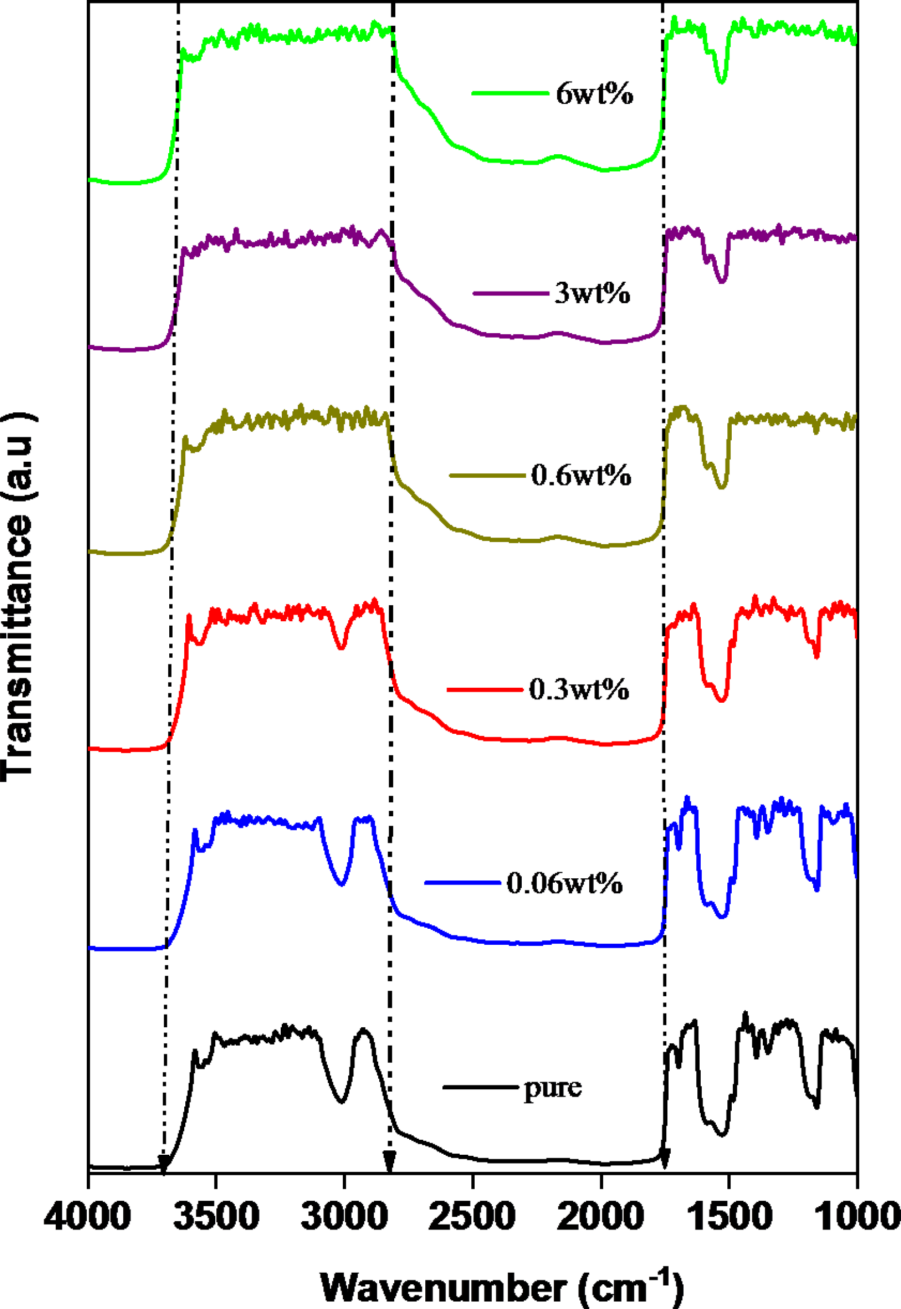
FT-IR spectra for Fe_2_O_3_/(PVA- PVP) films.

It is commonly known that the dopant effects on the structure of polymers can be identified using FT-IR spectroscopy. It offers details on how various polymeric film constituents interact with their structural components. Figure 2 displays FT-IR transmission spectra for Fe_2_O_3_ / (PVA/PVP) nanocomposites. Compared to the pure blend, the spectra display features of different stretching and bending vibration bands, in addition to modifications to specific band positions and alterations in intensity in others. Because of the hydroxyl (OH) stretching vibration, a strong broad absorption band was seen in the pure blend between 3600 and 2820 cm^− 1^^34,35,38^. This band shifts with nanocomposites as a result of Fe^+ 3^ interacting with the host polymer^39,40^.

The CH_2_ group caused asymmetric stretching at 2915 cm^− 1^, the C = C stretching vibration at 1656 cm^− 1 ,^ C = O stretching at 1700 and C-H bending at 3022 cm^− 1^ were detected in pure and low Fe_2_O_3_ content (see Table 1)^38^. However, at higher Fe_2_O_3_ content, the C-H bending will disappear. The observed drop in peak intensities over the complete FTIR spectrum could potentially be attributed to the intermolecular interaction between the blend matrix and Fe_2_O_3_ NPs. The peak position shifts and the intensity drops with the introduction of Fe_2_O_3_ NPs.

**Table 1:** FT-IR absorption spectra for Fe_2_O_3_ / (PVA- PVP) films.

Wavenumber (cm^− 1^)	Bond	References
3600–2821 cm^− 1^	O-H	^83^
1780 cm^− 1^	C = O	^84^
3022 cm^− 1^	C-H	^83^
1560 cm^− 1^	C = N	^29^

**Fig. 3:**
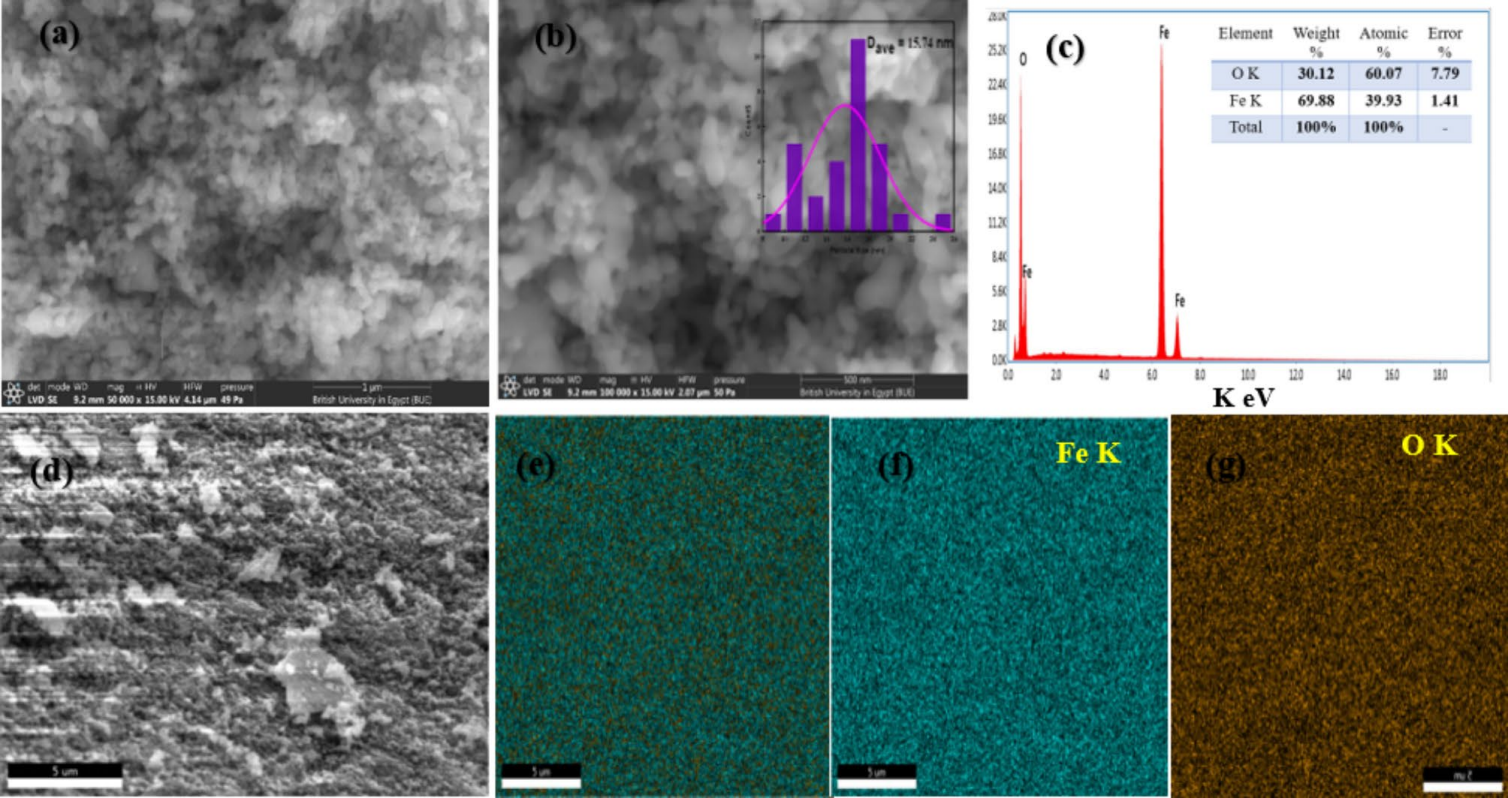
SEM-EDS elemental mapping of Fe K, O K composition where (**a**) electron image at 1 μm (**b**) electron image at 500 nm (**c**) weight% of various elements (**d**) Area EDS spectrum and right table for the atomic and. (**e**–**g**) analogous elemental mapping Fe K, O K elements.

The scanning electron microscope (SEM) is a highly efficient instrument for investigating the distribution and interaction of Fe_2_O_3_ nanoparticles. Figure 3a–c depicts agglomerated and stratified spherical nano-Fe_2_O_3_ has a diameter of 15.74 nm^41–43^. For all samples, elemental mapping using energetic dispersive x-ray spectrometry (SEM/EDS) was carried out. Individual sample chemical compositions from EDS testing have been compared to the specified chemical phase. Within the EDS analysis’s normal error range, the obtained compositions matched quite well. Figure 3d–g illustrates representative EDS spectra, elemental mapping, and secondary electron microscopy (EDS) at *x* = 5 μm. Various grains were tested to spot EDS to verify their chemical compositions. To verify sample homogeneity, elemental analysis for area EDS was performed (Fig. 3e–g). Figure 3d depicts an integrated area spectrum and tabulates the constituent elements’ atomic and weight percentages.

### Optical studies

**Fig. 4:**
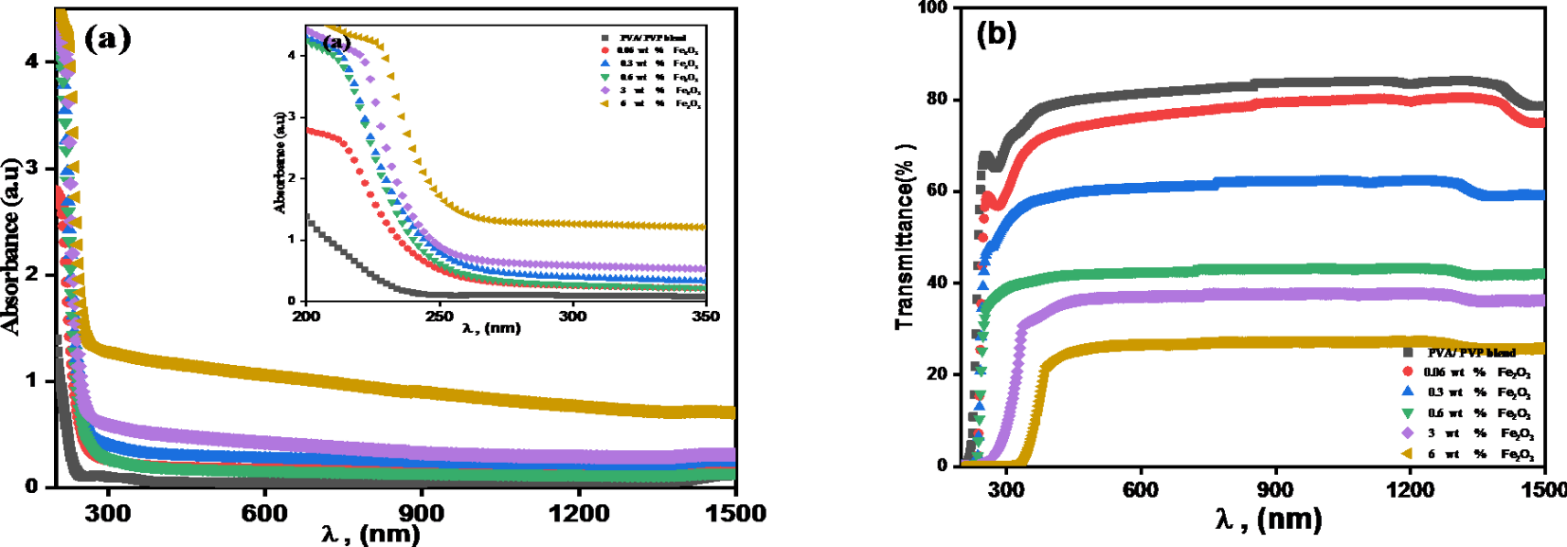
(**a**) Absorbance and (**b**) Transmittance plots for Fe_2_O_3_/(PVA- PVP) films with various concentrations of Fe^+ 3^ ions.

An important part of optical absorption research is analysing the structure and energy gap of the material. The transmission and absorption spectra for different Fe_2_O_3_ NPs doping concentrations in a hosting blend are shown in Fig. 4a and b, respectively. By adding more doped Fe_2_O_3_ NPs to the blend, it was possible to observe an improvement in the PVA/PVP blend’s absorption^44^.

Figure 4a displays the absorption spectra of the Fe_2_O_3_/(PVA/PVP) nanocomposites. The pure blend’s absorption spectrum has a sharp absorption edge^38^ which shifted as the concentration of Fe_2_O_3_ NPs in the polymer matrix rose. This shift to a higher wavelength was observed at the absorption edge 200 < *λ* < 350 nm (insert in Fig. 4a). This suggests that when the polymer matrix’s filler content rises, the optical band gap will decrease^15^. Figure 4b displays the optical transmission spectra of the (PVA/PVP)-Fe_2_O_3_ polymer nanocomposite films. From Fig. 4b, the polymer films exhibit negligible absorption between 350 and 1500 nm. Transmission drops in the 200–350 nm wavelength range in the UV region due to strong absorption. This happens due to the blend matrix’s Fe^+ 3^ -ion particle size increasing^39,40^. Pure blend exhibits the maximum optical transmission (roughly 80%). At a wavelength of about 700 nm, it drops to approximately 23% for the nanocomposite with 6 wt% Fe^+ 3^ NPs. Increasing Fe_2_O_3_ NPs percentage often reduces the optical transparency of the polymer blend. Fe_2_O_3_ NPs absorb and scatter light, reducing transparency and increasing opacity at larger concentrations.

Beer’s law is the theoretical foundation for the absorption coefficient, implying an exact relationship between the total amount of radiation absorbed and the quantity of absorbing molecules present in each sample^45^. Furthermore, the developed parameter provides important insights into the properties of unidentified energy bandgaps. The absorption coefficient *α* is represented by the equation below^46^:

3 $$\alpha =2.303({\rm A}/d)$$

where *d* represents the sample thickness, and *A* represents the absorbance. Reflectance (*R*) can be computed using the values of *T* and *A* in the following way^47^

4 $$R=1 - (T+A)$$

**Fig. 5:**
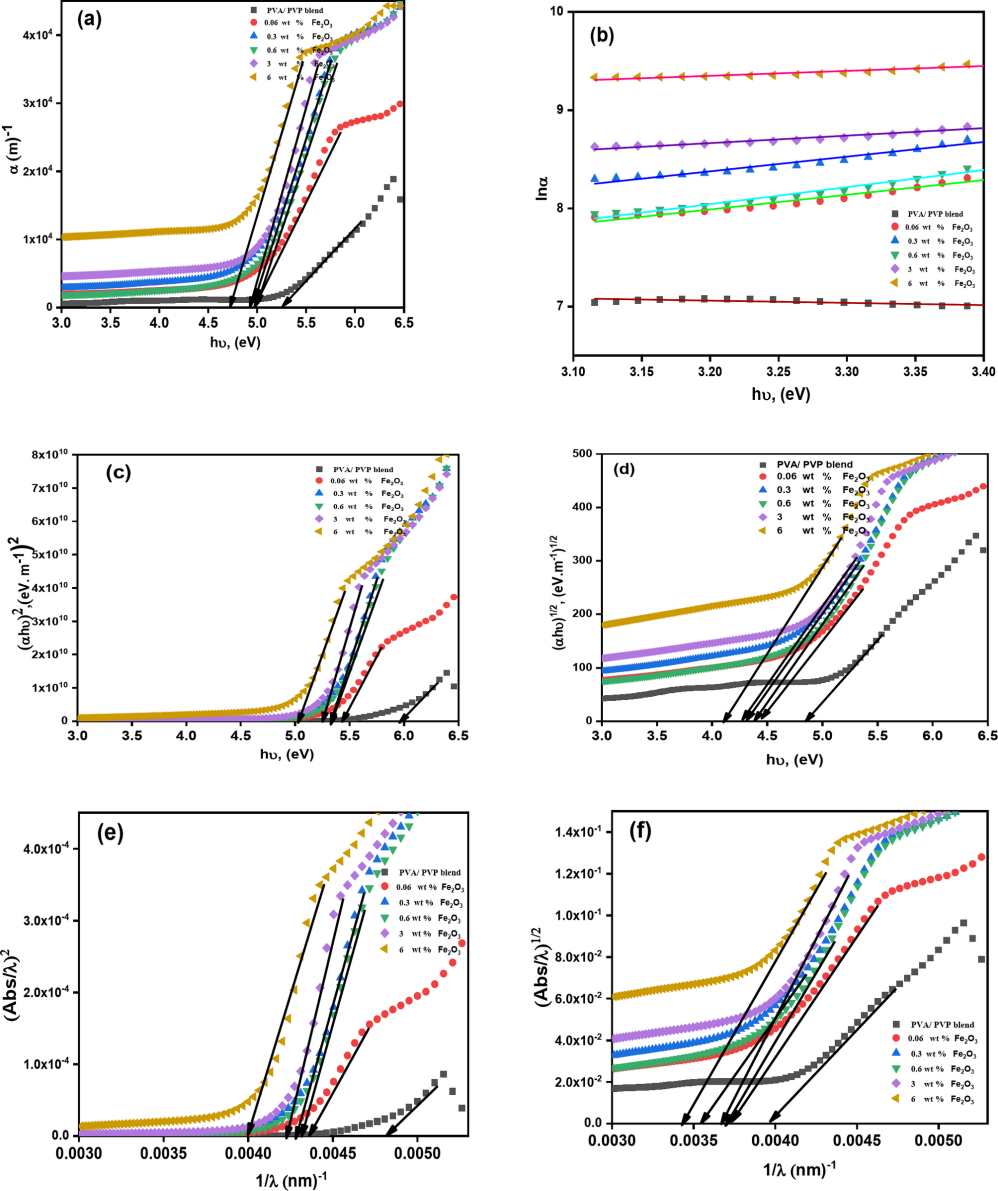
variation of (**a**) α, (**b**) lnα, (**c**) (αhν)^2^, (**d**) (αhν)^0.5^ versus hν and (**e**) (A/λ)^2^ versus (1/ λ), (**f**) (A/ λ)^1/2^ versus (1/ λ) for Fe_2_O_3_/ (PVA/PVP) films.

Figure 5a; Table 2 show the spectrum’s absorption edge value of (PVA-PVP) doped Fe^+ 3^ NPs nanocomposite films at varying concentrations. The measured and examined absorption coefficient data make it evident how the polymeric blend’s doping with (Fe_2_O_3_) has an impact. The suggested composites’ absorption coefficient values shifted to the less energetic range. when Fe^+ 3^ NPs were added. Consequently, it had an optical impact on the energy bandgap values. From Table 2, for the pure (PVA-PVP) polymer, the absorption coefficient was 5.33 eV, while for the 6 wt % Fe_2_O_3_ NPs, it was 4.71 eV. This optical result was confirmed Zyoud, S.H. et al.^16^ study of Nd^3+^-doped PVA/PVP composites, which showed that the doping level increased with a decrease in the polymeric films’ absorption coefficient.

**Table 2:** Optical energy gaps of Fe_2_O_3_ / (PVA- PVP) films.

Content of Fe_2_O_3_ doped in (PVA-PVP) (wt%)	E_edge_	E_g dir_	E_g ind_	$\:{E}_{dir}^{ASF}$	$\:{E}_{ind}^{ASF}$	E_U_ meV	
(PVA/PVP) blend	5.33	5.99	4.86	5.9	4.8	0.58	Present work
0.06 wt % Fe_2_O_3_	5.02	5.45	4.64	5.4	4.67	0.66	
0.3 wt % Fe_2_O_3_	4.99	5.37	4.6	5.34	4.69	0.67	
0.6 wt % Fe_2_O_3_	4.97	5.32	4.57	5.30	4.46	1.31	
3 wt % Fe_2_O_3_	4.92	5.24	4.53	5.22	4.33	2.01	
6 wt % Fe_2_O_3_	4.71	5.03	4.29	4.95	4.21	4.39	
(0:6 wt) PVA/ Fe_2_O_3_		(5.1:2.7)					^85^
(0:4Bi) PVA/PVP blend	(5.2:3.5)	(5.5:4.6)	(4.93: 4.2)		(5.02:3.0)		^85^
(0:5 wt) PVA/PVP/ZnO		(5.19:5.03)					^86^
(0–2wt) PVA/PVP/MgO		(5.2:4.8)					^87^
(0–4wt)PVA/PVP/CaZrO3		(5.0:3.70)					^88^
(0:5wt)Nb2O5		(5.52:4.79)	(4.48:3.1)		(4.55:3.10)		^89^

One can utilize Urbach’s tail width to investigate gaps in the unknown bandgap’s levels. The following equation represents the Urbach’s tail’s energy^16^:

5 $$\ln \alpha =\ln {\alpha _o}+\frac{{hv}}{{{{\rm E}_U}}}$$

Where $\:{E}_{U}$ is the Urbach’s tail’s energy, h is the Planck constant and $\:{\alpha\:}_{o}$ is the independent constant energy. Figure 5b was used to extract the computed $\:{E}_{U}$ values. It is important to emphasize how the composite films raised the $\:{E}_{U}$ value in contrast to the pure PVA/PVP blend polymer. The 6 wt % Fe_2_O_3_ NPs was influenced by the highest value of $\:{E}_{U}$ (4.39 meV). However, the rising values of $\:{E}_{U}$ with increasing doping concentration of Fe_2_O_3_ NPs, caused defects that altered the bandgap’s local state^48^. These defects indicate the degree of disordering in the samples. It’s important to note that the $\:{E}_{U}$ value takes into consideration the localized energy levels’ tail width in the material’s optical bandgap^49,50^. Comparable outcomes were documented in the literature^49,51,52^.

In crystalline and amorphous materials, the optical absorption spectra are a valuable tool for determining the optical band gap energy. The optical bandgap value can be estimated using the principal absorption resulting from electron excitation across the valence into the conduction band. Tauc’s equation is utilized to determine the samples’ bandgap, *E*_*g*_^16^:

6 $$\alpha h\upsilon ={\rm B}{\left( {h\upsilon - {{\rm E}_g}} \right)^r}$$

where *B* is a constant, h$\:\upsilon\:$ represents the photon energy, and r represents the power factor, having values of 0.5, and 2 for allowed direct, and indirect transitions, respectively. Dopants can alter the blend of polymers’ optical characteristics based on how they interact with the host matrix^53^. Figure 5c, d plot (*αhv*)^2^ and (*αhv*)^0.5^ values vs. the energy of the incident photon. The optical band gap energy (direct $\:{E}_{gd}$ and indirect $\:{E}_{gid}$) values are given from the extrapolated linear sections within the displayed curves to *hv* = 0. The resulting $\:{E}_{gd}$ and $\:{E}_{gid}\:$values of the pure and various ratios of Fe_2_O_3_ NPs incorporated PVA/PVP films are shown in Table 2. The values of $\:{E}_{gd}$ and $\:{E}_{gid}$ exhibit non-linear behavior as the Fe_2_O_3_ NPs doping percentage increases. Due to the energy levels in the films developing as the doping content increased, the films’ $\:{E}_{gd}$ and $\:{E}_{gid}$ decreased from 5.99 eV to 5.03 eV and from 4.86 eV to 4.29 eV, respectively. These defects are the principal source of charge transfer complex (CTC)-generation complex charging due to their interaction with PVA-PVP chains and Fe^+ 3^ -ion contents. These defects are responsible for the localized bandgap states. The formation of new energy levels (traps) from the HOMO to the LOMO is associated with an increase in the density of localized states in the bandgap, which decreases the values of $\:{E}_{g}$^54^. The same was determined by Choudhary and Sengwa^49,50^, who found that ZnO filling caused the pure blend’s $\:{E}_{gd}\:$to drop. The electronic structure of a material is directly influenced by its degree of crystallinity. More amorphous regions and lower crystallinity can introduce localized states that could lead to tailing in the absorption edge or a wider band gap (as noticed from XRD analysis).

The optical energy band gap^55^ was determined by using the absorption spectrum fitting (*ASF*) method, which was suggested by Souri et al. by making the following modifications to the Tauc model^56^:

7 $$\alpha (\lambda )=2.303{d^{ - 1}}{\rm A}(\lambda )=J{\left( {hc} \right)^{ - 1+m\lambda }}{\left( {{\lambda ^{ - 1}} - {\lambda _g}^{{ - 1}}} \right)^m}$$

Where *m* specifies the type of electronic transitions that cause absorption. The reciprocal values of *λ*_*g*_ were calculated by extrapolating the linear section of the plot of (*Aλ*^*−1*^) 1/m against *λ*^*−1*^ at zero. The energy gap was estimated by multiplying *λg*^*− 1*^ by 1240. Figure 5e, f shows how (Aλ^−1^)^0.5^ and (Aλ^−1^)^2^ fluctuate with λ^−1^ for X wt% of Fe_2_O_3_ NPs doped in PVA/PVP. Table 2 shows the values for $\:({\varvec{E}}_{\varvec{d}\varvec{i}\varvec{r}}^{\varvec{A}\varvec{S}\varvec{F}}$) and ($\:{\varvec{E}}_{\varvec{i}\varvec{n}\varvec{d}}^{\varvec{A}\varvec{S}\varvec{F}}$). Both direct ($\:{\varvec{E}}_{\varvec{d}\varvec{i}\varvec{r}}^{\varvec{A}\varvec{S}\varvec{F}}$), and indirect ($\:{\varvec{E}}_{\varvec{i}\varvec{n}\varvec{d}}^{\varvec{A}\varvec{S}\varvec{F}}$) decrease with increasing the content of Fe_2_O_3_ NPs in the polymer blend matrix due to introducing defects into a polymeric matrix^57^. This conclusion is consistent with the XRD data of the nanocomposites analyzed.

Blend films with Fe_2_O_3_ considerably impact the material’s extinction coefficient (*k*) and refractive index. Whereas the refractive index shows how light moves across a medium, the extinction coefficient computes how much light a substance absorbs. Fe_2_O_3_ NPs can modify the absorption and scattering characteristics of the material, which results in variations in *n* and *k*, and hence change the optical properties of blend films. The values of *n* from *R* and *k* can be calculated using the following formulas. The produced films’ *k* and *n* values are determined using the following Eqs^58–60^:

8 $$n=\frac{{\left( {1+{{\left( {R/4} \right)}^{0.5}}} \right)}}{{\left( {1 - {{\left( {R/4} \right)}^{0.5}}} \right)}}$$

9 $$k=\frac{{\alpha \lambda }}{{4\pi }}$$

**Fig. 6:**
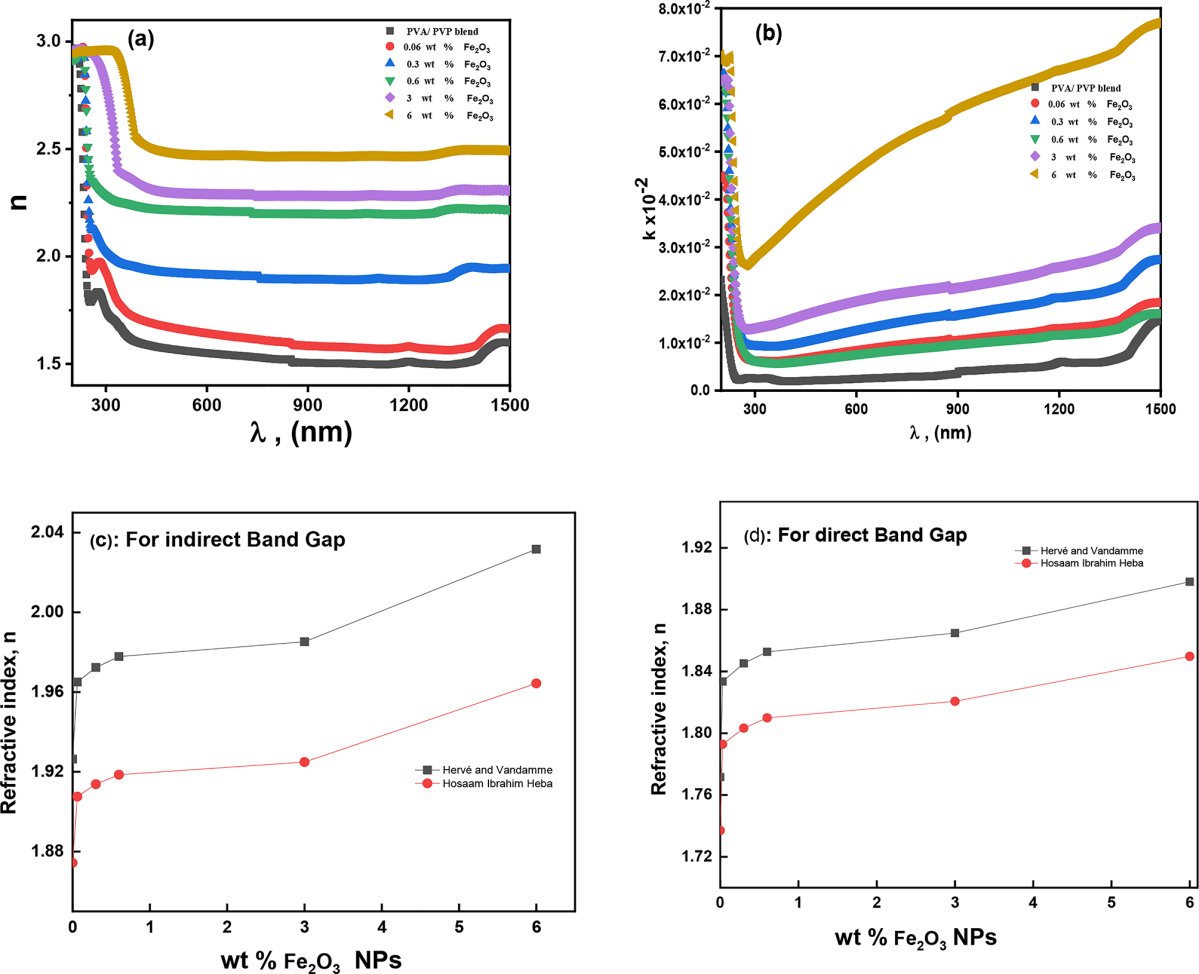
The variation of (**a**) n, (**b**) k versus the wavelengths, the computed refractive index (n) values using different model for both (**c**) indirect, (**d**) direct band gap for Fe_2_O_3_/(PVA-PVP) films.

Figure 6a shows the refractive index (*n*) vs. wavelength. The chart shows that the *n* values for the host blend increased as the concentration of Fe_2_O_3_ NPs rose. The rise in the *n* value suggested that doping had caused modifications to the intermolecular blend structure^61^, this enhanced the density and allowed for the creation of additional hydrogen bonds between blend chains. Adding 6 wt% Fe_2_O_3_ NPs to the mix matrix raises the refractive index from 1.56 to 2.47 (at λ = 500 nm). The *n* values decreased with increasing the *λ*, indicating that the blend molecules’ polarization state decreased because of the incident low-energy photons.

Figure 6b displays all blends’ extinction coefficient (*k*) data. The figure shows that as the concentration of Fe_2_O_3_ NPs rises, the extinction coefficient rises. The *k* value drops at low wavelengths in the 200–350 nm range. This could result from the incident photon carrying enough energy to excite the electron from one state to another. As shown in Fig. *8b, the *k* value likewise increases sharply at higher wavelengths (between 350 and 1500 nm), where the incident photon lacks the energy to excite the electrons. This resulted in a high extinction coefficient due to the energy lost. These outcomes agree with the earlier noted data^17,36^ .

The bandgap energy typically determines the photon absorption threshold in polymer composite materials. The transparency of a polymer composite film to the incident light beam is measured by its index of refraction (*n*). Thus, to comprehend the band structure of the polymer composite under investigation, a correlation between (*E*_*g*_) and (*n*) is important. The empirical methods put out by Hervé and Vandamme and Hosam-Ibrahim-Heba can be used to estimate the relationship between (*n*) and both indirect and direct optical band gaps $\:{E}_{gid}$ and indirect $\:{E}_{gd}$.

10 $$n = \sqrt {1 + {{\left( {\frac{{13.6}}{{{E_9} + 3.4}}} \right)}^2}} \;\;\;\;\;\;\left( {\text{Herv\'e}} \;\ {\text{and Vandamme eq}.} \right)$$

11 $$n=\sqrt {\frac{A}{{{E_g}^{{0.5}}}} - B} \;\;\left( {{\text{Hosam--Ibrahim--Heba eq}}.} \right)$$

Herein, *A* = 3.442, whereas *B* = √ 3.44.

The obtained *n* values are consistent with slight deviations (see Fig. 6c, d). The *n* value nearly ranged from 1.87 to 2.03 for indirect band transition and from 1.72 to 1.89 for direct band transition. The increase in (*n*) with increased NPs concentration can be attributed to the rise in the composite’s optical density and a decrease in both indirect and direct optical band gaps.

### **Optical limiting characterization**

**Fig. 7:**
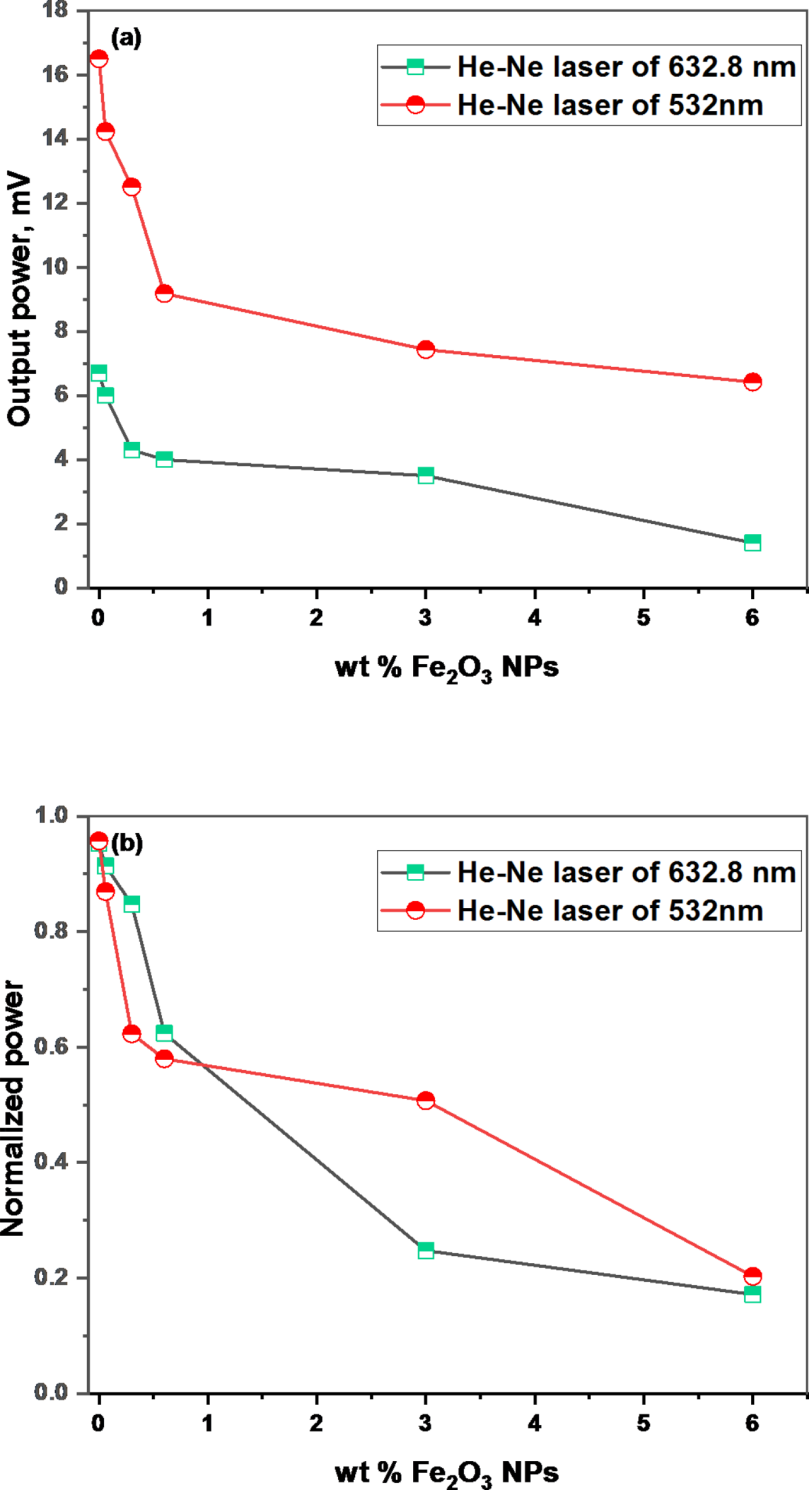
(**a**) Output power and (**b**) Normalized power for Fe_2_O_3_/ (PVA-PVP) films.

Devices intended to filter incident electromagnetic radiation are known as optical limiters^62^. Preventing laser degradation of optical sensors and components is a widely used application of this effect^30^. To quantify output power and normalized (output/input) power for investigating the optical limiting characteristics (OLC) for the films under investigation, two distinct laser sources (green and He-Ne laser sources) with wavelengths of 533 nm and 632.8 nm, as well, were used. Figure 7 displays the output power measurements and derived normalized power (Output/Input). Table 3 shows the optical limiting factors for each source. The source’s output powers are high for the pure blend PVA/PVP, but increasing Fe_2_O_3_ NPs content in the blend matrix decreases output power for lasers with 533 and 632.8 nm wavelengths. Increasing the Fe_2_O_3_ NPs content in the blend matrix to 6 wt % Fe_2_O_3_ NPs decreases the output power from 22.98 to 3.6 mW and 6.59 to 1.4 mW. As a result, the filler concentration has a noticeable impact on the OLC. The two sources’ different output power values can be attributed to the composite film’s sensitivity to light influx. In the blend matrix, a sample containing a higher concentration of Fe_2_O_3_ NPs has more molecules per unit volume, which takes part in optical interactions during nonlinear absorption mechanisms^62^. As a result, the examined films’ OLC is linked to the sample’s absorption and scattering capacities. The polymer sample with a blend of 6 wt% Fe_2_O_3_ NPs had the smallest normalized power value. Due to the high light power attenuation, this sample can be used as an optical limiting laser.

**Table 3:** Output power and normalized power for two laser sources, He-Ne at 638.2 nm and 532 nm for Fe_2_O_3_ / (PVA-PVP) films.

Films	Red laser 632 nm I_o_= 6.53mW		Green laser 532 nm I_o_= 22.98mW	
	Output power	Normalized power	Output power	Normalized power
(PVA/PVP) blend	6.7	0.95	20	0.95
0.06wt% Fe_2_O_3_	6	0.86	19.2	0.91
0.3wt% Fe_2_O_3_	4.3	0.62	17.8	0.85
0.6wt% Fe_2_O_3_	4	0.58	13.1	0.62
3wt% Fe_2_O_3_	3.5	0.51	5.2	0.25
6wt% Fe_2_O_3_	1.4	0.20	3.6	0.17

### Radiation shielding

Numerous methods exist for measuring the interaction between photons and an absorbing material as they pass through it. Generally, the transmission of incoming photon energy is governed by the modified Beer-Lambert equation^63^:

12 $$I={I_o}{e^{ - \mu x}},$$

**Fig. 8:**
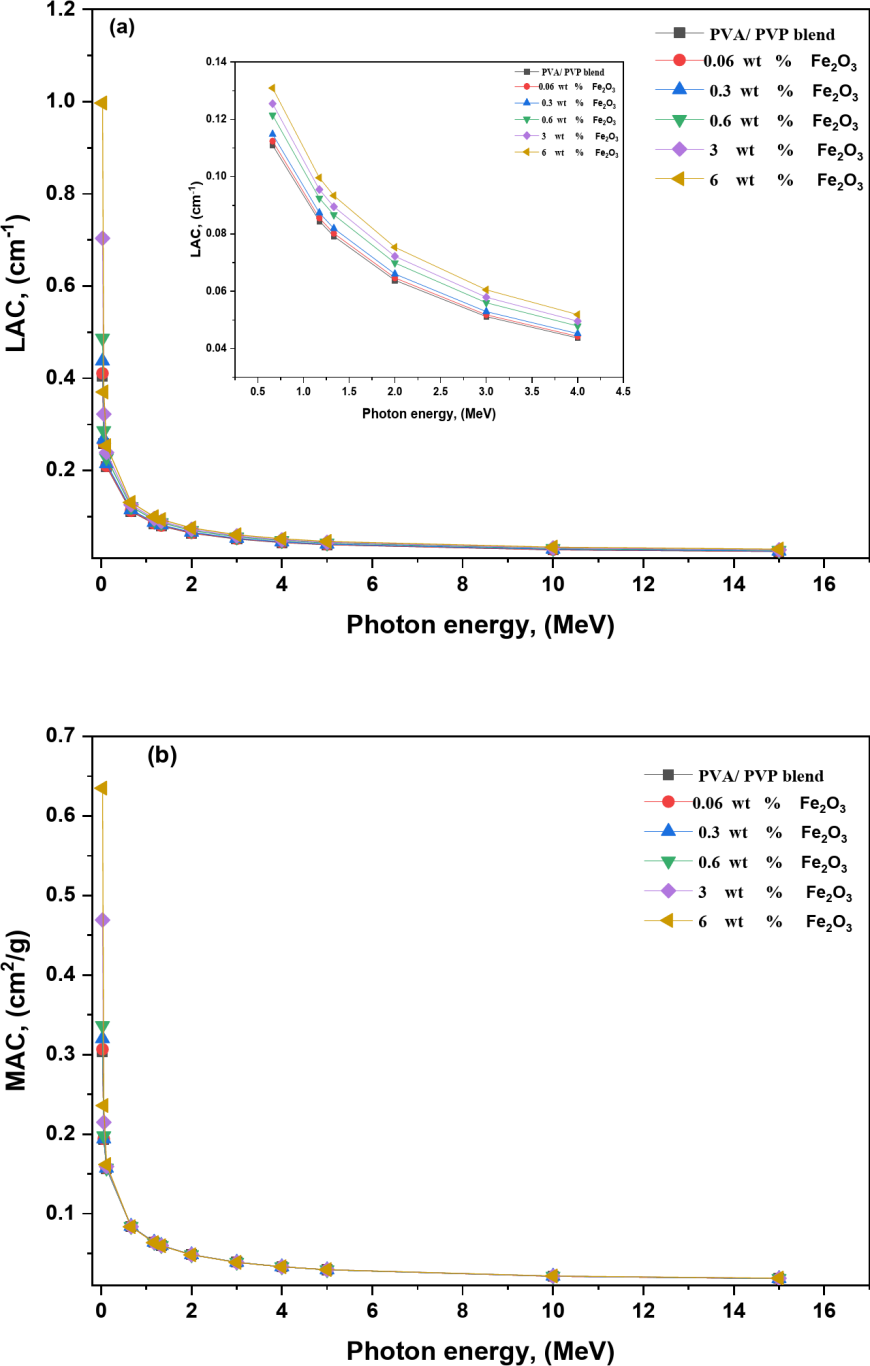
The variations of (**a**) linear attenuation coefficients and (**b**) mass attenuation coefficients of the Fe_2_O_3_/(PVA-PVP) films in the energy range 0.03–15 MeV.

Where *I* and $\:{I}_{o\:\:}$ are the intensity of the transmitted and incident monoenergetic photons, respectively, *µ* is the linear attenuation coefficient *LAC*, and *x* is the travelled distance in absorbing material. The absorber’s thickness, photon energy, and sample composition impact the *LAC (µ)* values. The gamma-ray attenuation properties of five composite samples reinforced with Fe_2_O_3_ NPs were evaluated in this study. NGCal software was used to calculate *LACs* for the samples. The film density increased from 1.32 g/cm^3^ to 1.57 g/cm^3^. Because of the highest concentration of Fe_2_O_3_ NPs in its structure, the sample containing 6 wt % Fe_2_O_3_ had the highest density. There is a relationship between density, the values of the attenuation coefficient, and the weight% of Fe_2_O_3_ NPs since the linear attenuation coefficient is a density-dependent feature. The linear attenuation coefficients (cm^− 1^) as a function of incident photon energy are displayed in Fig. 8a. As photon energy increased, the linear attenuation coefficients decreased significantly. The three main photon interaction cross-sections that could happen in the material are photoelectric effect *(PEE)*, Compton scattering *(CS)*, and pair production *(PP)*. The PNC films have the highest *LAC* values in the low-energy region, where the *PEE* dominates, and the cross-section area is proportional to the photon’s energy as 1/E^3.5^ and the PNC films’ atomic numbers *(Z*^*(4–5)*^*).* The difference between the *LAC* values for the PNC films begins to decrease at the medium energy range. This is because the influence of the *CS* rises in that range, resulting in a cross-section that is proportional to *Z* and reduces exponentially with energy. Further increases in photon energy E > 1.022 MeV cause a minor increase in *LAC* values because the *PP* is the dominant interaction, and the cross-section is proportional to Z^2^. Our data showed that the sample 6 wt % Fe_2_O_3_ NPs (*ρ* = 1.57 g/cm^3^), with the largest content of Fe_2_O_3_ NPs, had the maximum *LAC* values for all input photon energies. For example, at 3 MeV, the *LAC* values rose from 0.051 cm^− 1^ for the pure blend to 0.061 cm^− 1^ for 6 wt % Fe_2_O_3_.

The mass attenuation coefficient (*MAC(*$\:{\mu\:}_{m})$*)* may be calculated as follows^64^:

13 $$\:MAC=\frac{\mu\:}{\rho\:}=\:{\mu\:}_{m}$$

where *ρ* is the absorbing medium’s mass density. By using *MAC*, one can measure how different-energy photons interact with a substance. Figure 8b shows how the *MAC* varies with photon energy. When the energy was increased, the *MAC* values decreased. For example, as the energy increased from 0.662 to 1.332 MeV, the pure blend *MAC* value decreased from 0.083 to 0.059 cm^2^/g. In a prior study^65^, the *MAC* values for pure PVA decreased from 0.086 to 0.057 cm^2^/g as the energy rose from 0.662 to 1.332 MeV. Increasing the Fe_2_O_3_ content causes MAC values to slightly rise in the lower energy range, but this change is insignificant in the higher energy range. The *MAC* value for the pure blend was 0.193 cm^2^/g at 60 keV, and it climbed to 0.236 cm^2^/g as the Fe_2_O_3_ component reached 6 wt %. Shams et al.^66^, observed similar results, in PVA as the BaTiO_3_ concentration rose from 0.65 to 3.57 wt %, the *MAC* value increasing from 0.218 to 0.252 cm^2^/g at 81 keV.

A material’s capacity to absorb gamma rays can be expressed in terms of its half-value layer *HVL*, mean free path *MFP*, and tenth-value layer *TVL* values. Lower values for *HVL*, *MFP*, and *TVL* indicate higher radiation absorption efficiency. M. Rashad^67^ demonstrated this result using PVA with 1 wt% of Ni, NiO, and Fe_2_O_3_ nanoparticles.

The half-value layer (*HVL*) is the thickness required to reduce incident photon intensity to half of its original value. It can be calculated as follows^68,69^:

14 $$HVL=\frac{{\ln 2}}{\mu },$$

The tenth-value layer *TVL* that reduces the incident photon intensity to one-tenth of its original value is given by^70^:

15 $$TVL=\frac{{\ln 10}}{\mu },$$

The mean free path (*MFP*) of a photon is the average distance it travels before interacting with an absorbing material. This can be computed as follows^68^ :

16 $$MFP=\frac{1}{\mu },$$

**Fig. 9:**
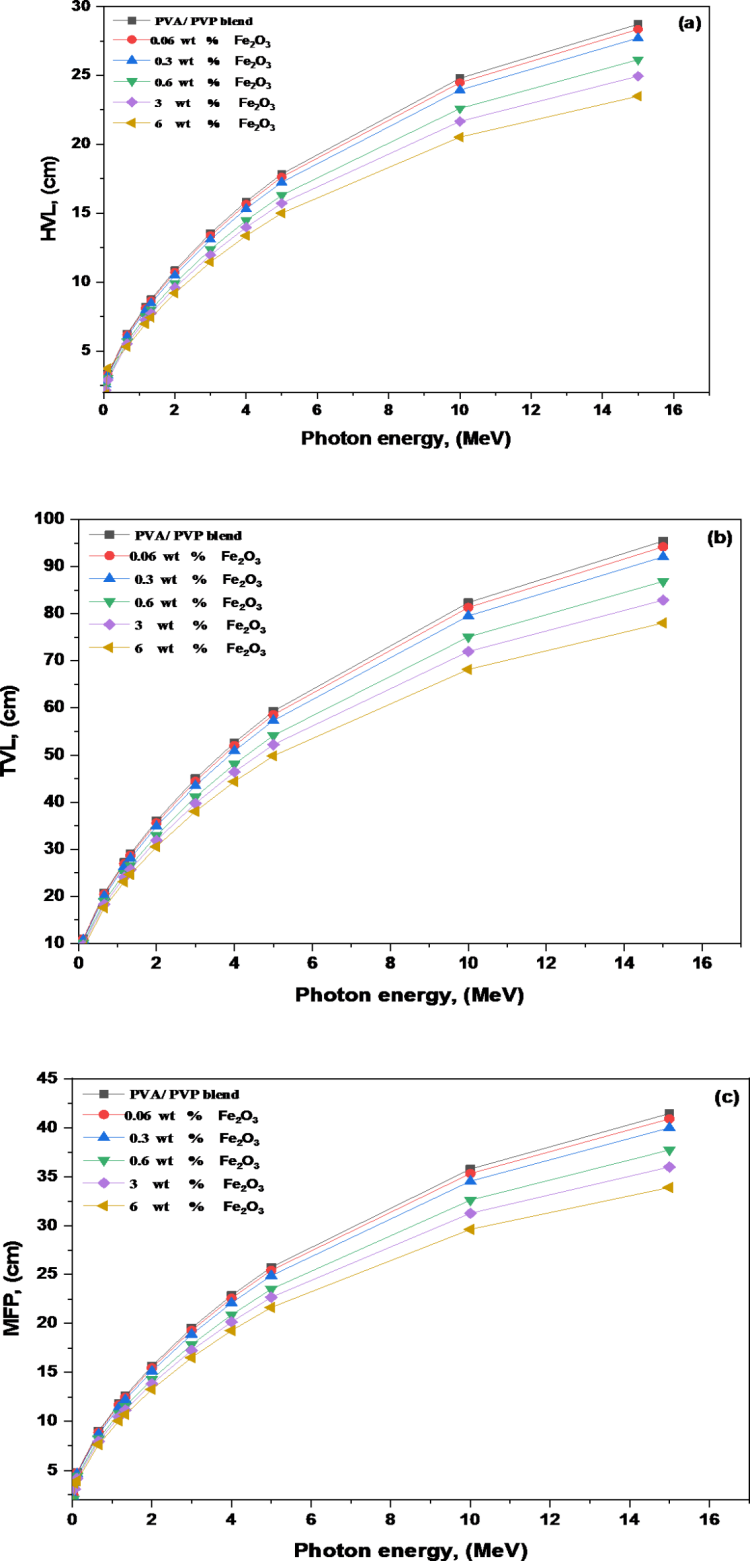
The variations of (**a**) The half value layer (*HVL*), (**b**) The tenth value layer (*TVL*), and (**c**) Mean free paths (*MFP*) for Fe_2_O_3_/(PVA-PVP) films in the energy range 0.03–15 MeV.

Figure 9a–c show how the *HVL*, *TVL*, and *MFP* values of the PNC films vary with the photon energy. As photon energy rises, so do the *HVL*, *TVL*, and *MFP* values. Figure 9a shows that when the energy increases from 3 to 10 MeV, the pure blend *HVL* value drops from 13.55 to 24.79 cm. The *HVL*, *TVL*, and *MFP* values were reduced as the Fe_2_O_3_ content rose. For instance, at 2 MeV the *HVL* for a pure blend is 10.85 cm; when the Fe_2_O_3_ content increases to 6 wt %, this value drops to 9.19 cm. Shams et al. previously reported similar results when examining PVA doped with BaTiO_3_^66^. For 6 wt % Fe_2_O_3_ nanocomposite film has the lowest *HVL* and *TVL* due to its higher density and higher *LAC*, which indicates that it can absorb more ionizing radiation than the other PNC films.

### Fe_2_O_3_/(PVA-PVP) polymeric films’ stress-strain behavior

**Fig. 10:**
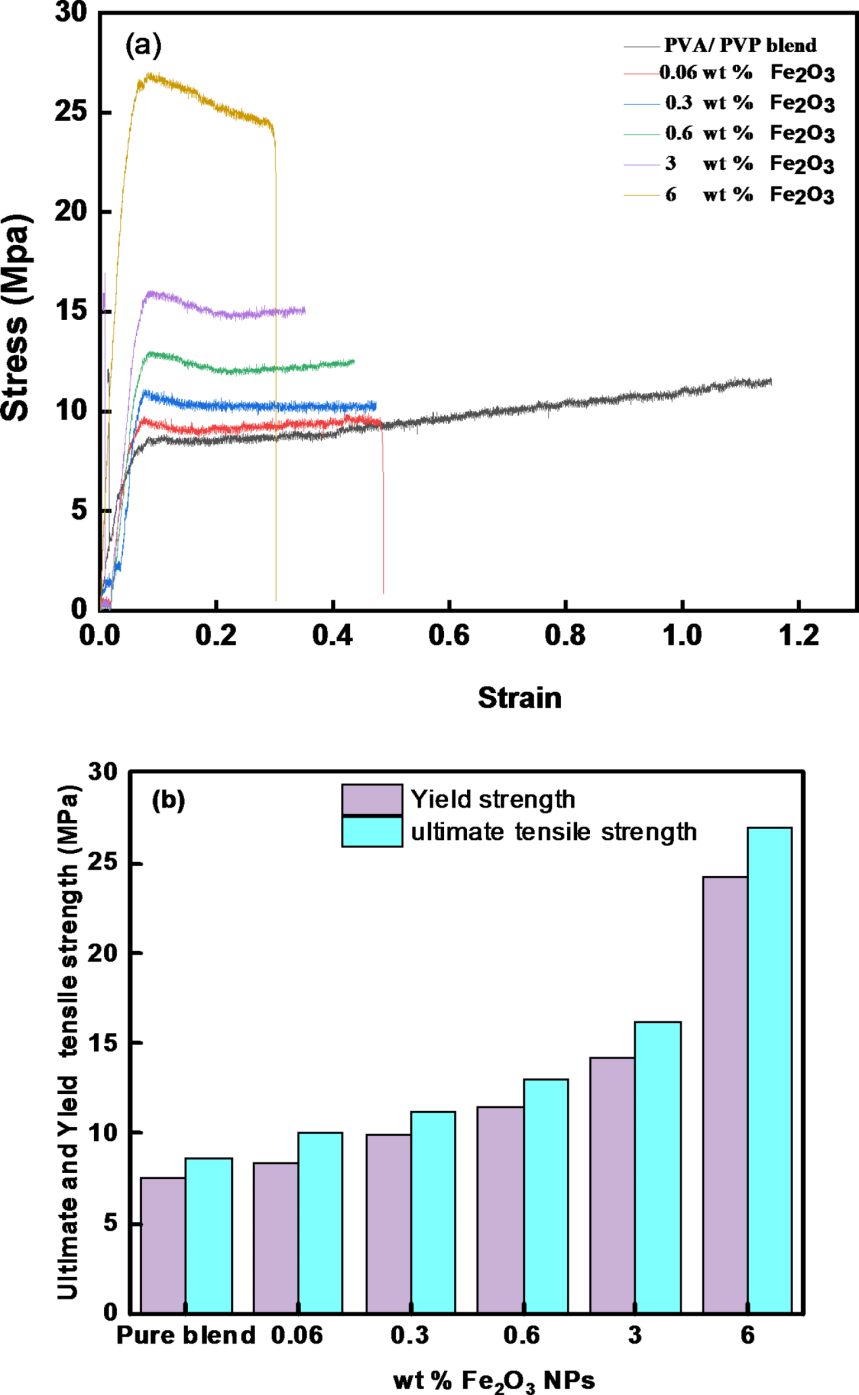
(**a**) The representative stress-strain curves (**b**) Yield strength (Young’s modulus), and Ultimate tensile Strength at a strain rate of 1.96 × 10^− 3^ sec^− 1^ at room temperature for Fe_2_O_3_/(PVA-PVP) films.

Mechanical factors greatly influence the quality and performance of films. In Fig. 10, the stress-strain curves of Fe_2_O_3_/(PVA-PVP) films are illustrated at a strain rate of 1.96 × 10^− 3^ sec^− 1^ at room temperature. The mechanical properties of Fe_2_O_3_/PVA-PVP polymeric nanocomposite films are summarized in Table 4. The mechanical characteristics of the composites are significantly affected by the Fe_2_O_3_ content. A typical stress-strain graph is shown in Fig. 10a, where films made of (PVA-PVP) blend exhibit high elongation at break but low strength^71^. The nanocomposite films’ yield strength, tensile strength, and Young’s modulus (see Fig. 10b, c) were greatly enhanced with increased Fe_2_O_3_ content. Compared to pure (PVA-PVP) films, nanocomposite films display higher tensile strength and elongation at break. For instance, the tensile strength of the composite film containing 6% Fe_2_O_3_ is measured at 27.03 MPa, a significant improvement from the 8.66 MPa of the pure (PVA-PVP) film. However, as the concentration of the doping increased, the films’ equivalent elongation at break decreased, these findings indicate that Fe_2_O_3_ NPs effectively enhanced composite films^37,72^. By effectively embedding (PVA-PVP) on Fe_2_O_3_ NPs, interactions between matrix and filler can be significantly improved, facilitating stress transfer under external strain^37,72^.

**Table 4:** The computed mechanical properties for Fe_2_O_3_ /(PVA-PVP) films.

Samples	Young’s modulus Y	Elongation at break, (ε %)	Tensile Strength (T.S) (MPa)	Growth percentage	Reference
(PVA/PVP) blend	1.43	1.14	8.66	–	Present work
0.06wt% Fe_2_O_3_	1.59	0.48	10	15.47	
0.3wt% Fe_2_O_3_	0.79	0.46	11.19	29.21	
0.6wt% Fe_2_O_3_	1.71	0.43	13	50.11	
3wt% Fe_2_O_3_	2.12	0.34	16.2	87.06	
6wt% Fe_2_O_3_	5.02	0.29	27.03	212.12	
PVA/Fe_2_O_3_ nanocomposites	From 1.4 to 11.1	From 39.8 to 14.6	From 85.2 to 117.4		^85^

The degree of Fe_2_O_3_ NPs in (PVA/PVP) matrix interfacial bonding may be responsible for the differences in mechanical properties. Disperse more uniformly, improving load transmission and thereby increasing tensile strength. Fe_2_O_3_ NPs significantly impact the PVA/PVP blend’s microstructure and crystallinity. As can be observed from the XRD, FTIR, and SEM images, varying concentrations of Fe_2_O_3_ have different effects on the crystallization process, which results in changes in mechanical characteristics. The improvement in mechanical qualities can be quantified by calculating the growth percentage compared to pure PVA/PVP. For each concentration, the growth percentage in tensile strength relative to the pure blend can be calculated as:

17 $$Growth.Percentage=(\frac{{(Tensile.Strength.(withF{e_2}{O_3}))}}{{Tensile.Strength(purePVA/PVP)}} - 1) \times 100$$

The PVA/PVP nanocomposites’ ability to balance strength and elasticity is improved by the addition of 6 wt% Fe_2_O_3_ NPs. The growth percentage calculation would provide additional insight into how each concentration compares against the pure blend, facilitating a more comprehensive comprehension of the Fe_2_O_3_ reinforcement’s efficacy (see Table 4). This finding is consistent with the previous study on enhancing some polymers using a certain Fe_2_O_3_ NPs ratio^73,74^.

### Dielectric behaviour studies

**Fig. 11:**
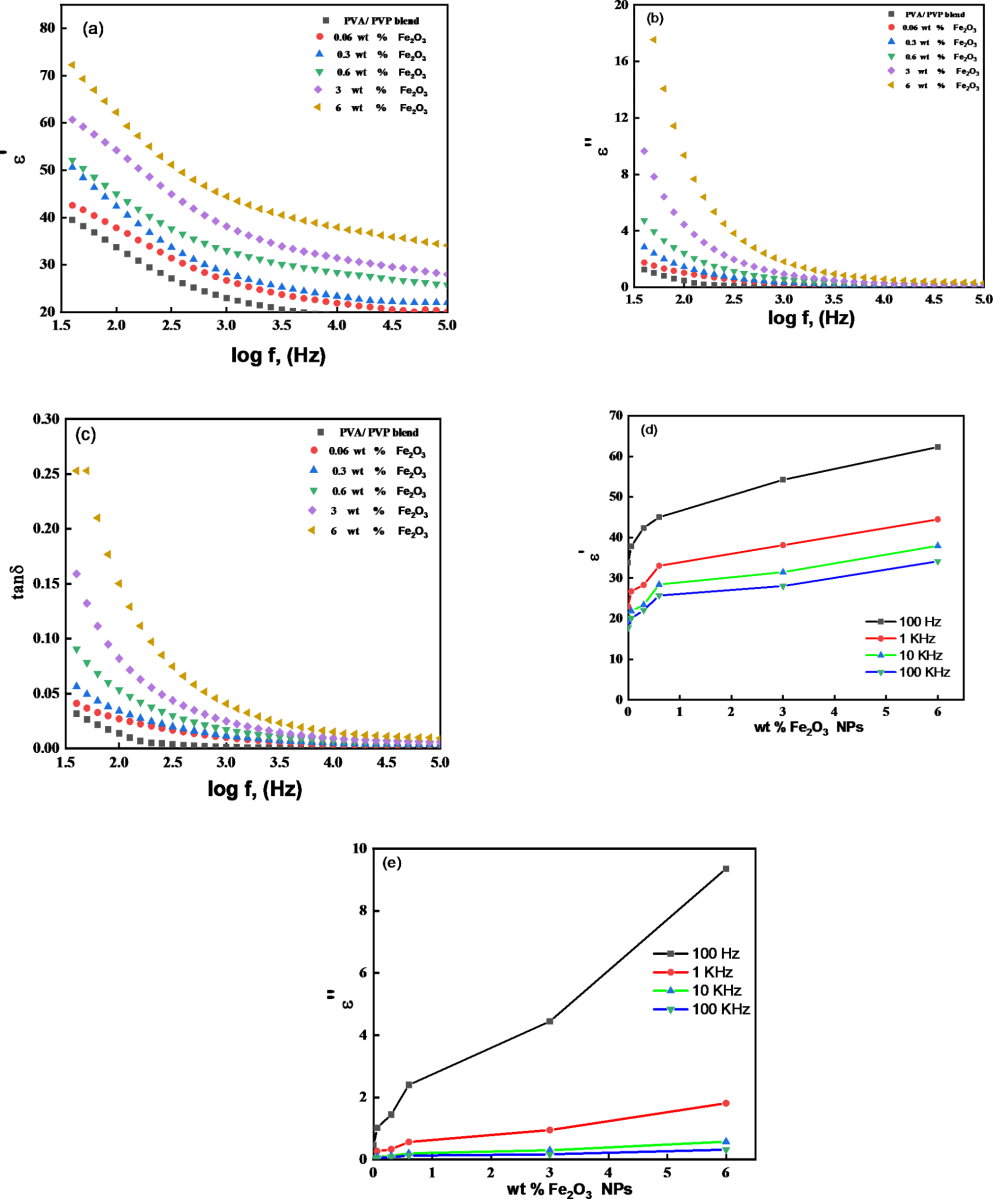
Frequency dependent (**a**) dielectric constant $\:{\epsilon\:}^{{\prime\:}}$, (**b**) dielectric loss $\:{\epsilon\:}^{{\prime\:}{\prime\:}}$, and (**c**) tanδ, and (**d**,**e**) $\:{\epsilon\:}^{{\prime\:}}$ and $\:{\epsilon\:}^{{\prime\:}{\prime\:}}$ values for Fe_2_O_3_ concentration dependent for Fe_2_O_3_/(PVA-PVP) films at 30^o^C .

An efficient method for learning about conduction and relaxation mechanisms is to test the dielectric properties of polymeric materials. The formulas^54^ below have been used to determine the dielectric constant $\:{\epsilon\:}^{{\prime\:}}$ and dielectric loss $\:{\epsilon\:}^{{\prime\:}{\prime\:}}$:

18 $${\varepsilon ^\prime }=\frac{{C\left( F \right).d\left( m \right)}}{{{\varepsilon _o}\left( {F.{m^{ - 1}}} \right).A\left( {{m^2}} \right)}},$$

where *C*, *d*, $\:{\epsilon\:}_{0}$, and *A* is the film capacitance, the film thickness, the space permittivity ($\:{\epsilon\:}_{0}$=8.85 × 10 ^− 12^ Fm ^− 1^) and the electrode area, respectively. The complex dielectric permittivity ($\:{\epsilon\:}^{{\prime\:}}$ and $\:{\epsilon\:}^{{\prime\:}{\prime\:}}$) and $\:\text{tan}\delta\:$ spectra of the produced films at 30 ^o^C are displayed in Fig. 11a–c based on the findings. These spectra reveal a characteristic of PNC materials: a decrease in the values of $\:{\epsilon\:}^{{\prime\:}}$ in the PNC film as the frequency increases.

Figure 11a, b demonstrates how both real and imaginary parts of the complex permittivity of Fe_2_O_3_ /(PVP-PVA) films at ambient temperature fluctuate with frequency. Electrode polarisation at lower frequencies is attributed to the high dielectric constant range, which occurs from the accumulation of charges on an electrode^75^. The rapid periodic reversal of the electric field at high frequencies is responsible for the dielectric constant decrease because it prevents charge accumulation, and, as a result, no polarization effect is seen. As frequency increases, complex permittivity’s real and imaginary parts also decrease. Additionally, when the content of Fe_2_O_3_ in polymeric blends increased, the dielectric constant ($\:{\epsilon\:}^{{\prime\:}}$) and dielectric loss ($\:{\epsilon\:}^{{\prime\:}{\prime\:}}$) values in all PNC films moderately shifted towards the lower side. The PNC based on 6 wt% nanofiller exhibits the highest conductivity and dielectric constant^76^ due to an increase in the quantity of free mobile charges for transportation.

Other ways to express dielectric loss include in terms of the loss tangent (*δ*), which is shown as

19 $$\tan \delta ={\varepsilon ^{\prime \prime }}/{\varepsilon ^\prime }$$

The fluctuation of tan *δ* with frequency for (PVP-PVA) xwt% Fe_2_O_3_ at room temperature with different amounts of nano-fillers is displayed in Fig. 11c. This Figure shows that tan δ decreases with increasing frequency. This could be caused by polarization (MWS effect). Another possible explanation is that dipoles have begun to respond to field variations at higher frequencies. Tan δ increases with Fe_2_O_3_ concentration in PVA/PVP nanocomposites^77,78^.

Plotting the$\:\:{\epsilon\:}^{{\prime\:}}$ and $\:{\epsilon\:}^{{\prime\:}{\prime\:}}$ values of the PNC films against wt% Fe_2_O_3_ NPs at set frequencies are shown in Fig. 11d, e, which provides an understanding of the behavior of the films with Fe_2_O_3_ NPs concentration. The PVA–PVP blend’s dispersion of Fe_2_O_3_ significantly raises the $\:{\epsilon\:}^{{\prime\:}}$ values. The correlation observed between the PNC film $\:{\epsilon\:}^{{\prime\:}}$ and $\:{\epsilon\:}^{{\prime\:}{\prime\:}}$ values and the concentration of Fe_2_O_3_ NPs imply that Fe_2_O_3_ NPs significantly increase the parallel dipolar ordering of the –OH and C = O functional groups when they interact with the PVA–PVP structure. These results imply that Fe_2_O_3_ NPs should be loaded into a PVA–PVP blend matrix (70/30 wt %) to get the optimum tunable dielectric properties and be used in low loss nanocomposite materials^76^.

By examining the modulus ($\:{M}^{*}$), the loss tangent and dielectric permittivity analysis are investigated further. The primary benefit of modulus analysis is its capacity to isolate the polarization effect from the bulk relaxation occasion in the polymer electrolyte^43^. The following equations connect the complex permittivity ($\:{\epsilon\:}^{*}$) to the complex electric modulus ($\:{M}^{\text{*}}$):

20 $${M^ * }=\frac{1}{{{\varepsilon ^ * }}}={M^\prime }+i{M^{\prime \prime }}$$

where the real component is

21 $${M^\prime }=(\frac{{{\varepsilon ^\prime }}}{{{\varepsilon ^{\prime 2}}+{\varepsilon ^{\prime \prime 2}}}})$$

and the imaginary component is

22 $${M^{\prime \prime }}=(\frac{{{\varepsilon ^{\prime \prime }}}}{{{\varepsilon ^{\prime 2}}+{\varepsilon ^{\prime \prime 2}}}})$$

**Fig. 12:**
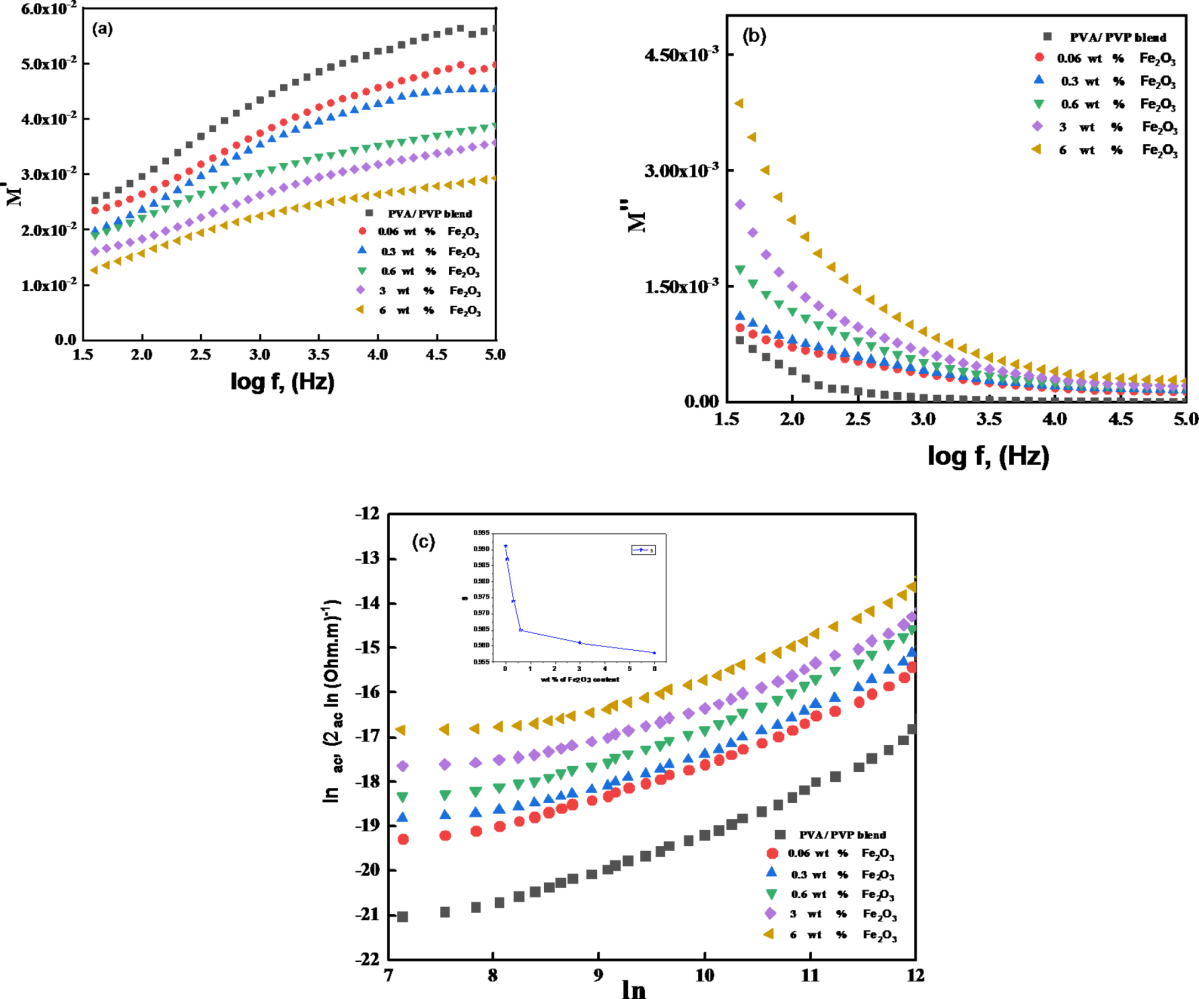
(**a**,**b**)Plots of M$\:{\prime\:}$ and M$\:{\prime\:}{\prime\:}$ values and (**c**) Frequency-dependent ac electrical conductivity for Fe_2_O_3_ /(PVA–PVP) films at 30^o^C.

Where $\:{M}^{{\prime\:}}$ denotes the real part of the dielectric modulus and $\:{M}^{{\prime\:}{\prime\:}}$ is its imaginary part. For (PVA–PVP) / Fe_2_O_3_ nanocomposites, the real part ′ (*ω*) and loss part $\:{M}^{{\prime\:}{\prime\:}}$ (*ω*) spectra in Fig. 12a, b, respectively, indicate the electric modulus at 30 °C. These PNC materials’ M′ (Fig. 12a) values rapidly increase with increasing frequency in the lower frequency range^77^. This study investigates the effects of Fe_2_O_3_ concentration on organic-inorganic composites’ electrical and dielectric characteristics. PVA/PVP blend matrix basically forms a variety of micro-environments related to hydrogen bonding between PVA-PVA chains in the crystalline and amorphous phases, PVA-PVP chains in the amorphous phase, and at the amorphous–crystalline interface^78^.

The $\:{M}^{{\prime\:}{\prime\:}}$values of the Fe_2_O_3_ doped PVA/PVP composite films are displayed in Fig. 12b. For every synthetic polymeric composite, the $\:{M}^{{\prime\:}{\prime\:}}$and $\:{\epsilon\:}^{{\prime\:}{\prime\:}}$ spectra exhibited the same behavior. Furthermore, $\:{M}^{{\prime\:}{\prime\:}}$values rose with increasing Fe_2_O_3_ doping levels on the host PVA/PVP polymer but decreased with increasing frequency. These findings thus far pointed to a notable shift in the direction of a shorter relaxation period and higher ionic conductivity^79^.

The fundamental features of ion dynamics can be properly identified by computing the frequency exponent(s) based on Jonscher’s well-known universal power low as follows^80^:

23 $${\sigma _{ac}}\left( \omega \right)={\sigma _{total.ac}}\left( \omega \right) - {\sigma _{dc}}\left( {\omega =0} \right),$$

24 $${\sigma _{dc}}\left( \omega \right)=B{\omega ^s}$$.

where *σ*_*dc*,_*ω*,* B*, and *s* are the dc conductivity, angular frequency, Boltzmann’s constant in eV, and the frequency exponent of the polymer films. The frequency dependence of the electrical conductivity ($\:{\sigma\:}_{ac}$) of polymeric films at 30 °C is shown in Fig. 12c. As a result of electrostatic interactions between the polymer and Fe_2_O_3_ NPs, the$\:{\:\:\sigma\:}_{ac}$ values of these materials improve, indicating an increase in the number of free charges and/or the production of certain useful charge conductive links^81^. In order to estimate the dc electrical conductivity ($\:{\sigma\:}_{dc}$) and s values for the prepared films with its Fe_2_O_3_ NPs content variation, a low-frequency $\:{\sigma\:}_{ac}$ data linear fit for electrical conduction ($\:{\sigma\:}_{dc}$) and a power law fit were applied, $\:{\sigma\:}_{ac}\left(\omega\:\right)={\sigma\:}_{dc}+B{\omega\:}^{s}$of high-frequency $\:{\sigma\:}_{ac}$ data. The s values ranged from 0.991 to 0.958 (see Table 5) demonstrating that the correlated barrier hopping (CBH) model, typical of disordered materials, describes the mechanism by which charge is transported in these nanocomposites^82^.

**Table 5:** Values of Dc electrical conductivity σ_dc_ obtained from the lower frequency fit of σ _ac_ spectra to the power law, and their consistent fractional exponent s at room temperatures T.

Samples	Ϭ_DC_	s
(PVA/PVP) blend	3.11 × 10^− 12^	0.991
0.06wt% Fe_2_O_3_	2.77 × 10^− 11^	0.987
0.3wt% Fe_2_O_3_	4.32 × 10^− 11^	0.974
0.6wt% Fe_2_O_3_	6.76 × 10^− 11^	0.965
3wt% Fe_2_O_3_	2.22 × 10^− 10^	0.961
6wt% Fe_2_O_3_	5.26 × 10^− 10^	0.958

## Conclusion

This work aimed to improve the gamma-ray shielding efficacy, as well as the optical, mechanical, and dielectric properties of PVA-PVP-based nanocomposites, by introducing different concentrations of Fe_2_O_3_ nanofillers. The findings indicate that the research objectives were attained. XRD revealed the rising amorphic of the nanocomposites. The PVA-PVP/0.6wt% Fe_2_O_3_ nanocomposite film grains have a homogeneous distribution of regular particles with an average size of about 16 nm. The direct and indirect bandgaps were calculated using ASF and Tauc’s equation, with somewhat lower values obtained at high doping concentrations. The films’ $\:{E}_{gd}$ and $\:{E}_{gid}$ values dropped from 5.89 eV to 5.03 eV and 4.86 eV to 4.29 eV, respectively, due to increased localized states (Urbach’s energy) near the HOMO and LUMO bands. The optical limiting of the films examined using He-Ne and green lasers was improved for the PVA/PVP blend with high Fe_2_O_3_ NPs. The inclusion of Fe_2_O_3_ affects the dielectric’s real and imaginary components. AC conductivity for blended PVA/PVP follows the Jonscher fitting. Frequency exponent parameter values show interactions between charge carriers during the hopping process of the correlated barrier hopping model (CBH). As the concentration of Fe_2_O_3_ increased so did *LAC*. *HVL*, and *MFP* values were lowest in the (PVA-PVP)/6 wt% Fe_2_O_3_. These findings suggested that the new composite films be used for a variety of applications, including optical protection, optoelectronics, and gamma shielding.

## Data Availability

All data that support the findings of this study are included within the article.
